# Treatment as Prevention and HIV Transmission in the US

**DOI:** 10.1001/jamanetworkopen.2026.30330

**Published:** 2026-08-21

**Authors:** Laura Matrajt, Sarah E. Stansfield, Mia Moore, Deborah J. Donnell, Elizabeth R. Brown, Myron S. Cohen, Dobromir Dimitrov

**Affiliations:** 1 Vaccine and Infectious Disease Division, Fred Hutchinson Cancer Center, Seattle, Washington; 2 Department of Applied Mathematics, University of Washington, Seattle; 3 Department of Global Health, University of Washington, Seattle; 4 University of North Carolina at Chapel Hill, Chapel Hill

## Abstract

**Importance:**

Providing treatment to all people living with HIV (PLHIV), a strategy known as treatment as prevention, is a highly effective intervention against HIV. Policies that reduce access to antiretroviral therapy (ART), an important component of treatment-as-prevention (TasP) strategies, might undermine decades of HIV control in the US and result in thousands of new HIV infections.

**Objective:**

To estimate the association between TasP and the HIV epidemic in the US from 2011 to 2025 and to project the outcomes of a potential rollback of this policy.

**Design, Setting, and Participants:**

In this decision analytical model, HIV incidence and prevalence from 2011 to 2030 were simulated. PLHIV were stratified by transmission group (men who have sex with men, heterosexual men, heterosexual women, and people who inject drugs) and stage of the HIV care cascade (undiagnosed, diagnosed but not receiving ART, receiving ART with detectable viremia, and receiving ART and virally suppressed).

**Exposure:**

Change in HIV care cascade.

**Main Outcomes and Measures:**

Annual HIV transmissions from 2011 to 2025 were compared with HIV care cascade (1) following the observed trend (with TasP) or (2) fixed at 2011 levels, without TasP. The impact of preexposure prophylaxis (PreP) rollout since 2017 was also estimated. Finally, HIV transmission from 2026 to 2030 was projected assuming that (1) 10% to 30% of PLHIV immediately lose ART access, reflecting the estimated share of ART funded through federal subsidies, (2) no change, or (3) a moderate increase in ART access.

**Results:**

In 2011, there were 1 008 300 PLHIV. In 2025, treatment as prevention was associated with an estimated 44.5% reduction in HIV incidence; PrEP, a 20.5% reduction; and treatment as prevention plus PrEP, a 61.8% reduction. From 2011 to 2025, TasP averted an estimated 197 400 new HIV acquisitions in the US, PrEP averted 33 200, and the 2 combined averted 293 200. Immediate losses of ART access affecting 10%, 20%, or 30% of treated PLHIV were associated with approximately 35 900, 81 700, and 111 200 additional HIV acquisitions between 2026 and 2030, respectively, substantially reversing the population-level gains achieved through TasP.

**Conclusions and Relevance:**

In this decision analytical model estimating the potential effects of reductions in ART access, findings suggest that these reductions will not only jeopardize individual health outcomes and over a decade of progress toward HIV elimination but also result in thousands of new HIV acquisitions over the next 5 years.

## Key Points

**Question:**

How was treatment as prevention (TasP) associated with the HIV epidemic in the US from 2011 to 2025, and what outcomes would rolling back this policy have?

**Findings:**

This decision analytical model estimated that TasP was associated with a 44.5% reduction in 2025 HIV incidence, averting an estimated 197 400 new HIV acquisitions since 2011. Immediately reducing treatment access by 20% would result in an estimated 80 000 additional HIV acquisitions between 2026 and 2030.

**Meaning:**

These findings suggest that reducing ART subsidies could result in thousands of new HIV acquisitions, undermining a decade of HIV epidemic control in the US.

## Teaser

This decision analytical modeling study evaluates the association of availability of HIV treatment with annual HIV transmissions in the US from 2011 to 2025 and estimates transmissions from 2026 to 2030 under different scenarios of access to antiretroviral therapy.

## Introduction

In 2011, a landmark clinical trial demonstrated that antiretroviral therapy (ART) could be used not only to prevent AIDS but also to substantially reduce sexual transmission of HIV.^1^ Follow-up studies^2,3,4^ reported no sexual transmission when a sexual partner was virally suppressed. These findings led to major changes in national and international HIV programs, fueling the rapid implementation of the undetectable = untransmittable (U=U) principle. Treatment recommendations were expanded to all people living with HIV (PLHIV), regardless of CD4 cell count.^5,6^ Treatment as prevention (TasP), defined as ART for the purposes of this study, has since become one of the primary strategies, alongside preexposure prophylaxis (PrEP), for controlling and mitigating the HIV epidemic worldwide and is a central pillar of the US Ending the HIV Epidemic initiative launched in 2019.^7,8^

In early 2026, news reports indicated that more than 20 US states were cutting or planning to cut AIDS drug assistance programs (ADAP), which provide subsidies for vital ART medications for 25% of PLHIV.^9^ Given the effectiveness of TasP, these cuts will likely lead to thousands of new HIV acquisitions. Here, we use counterfactual modeling to estimate the outcomes associated with TasP and their association with HIV incidence and prevalence in the US from 2011 to 2025, and project the effect of the potential rollback of these policies starting in 2026. We hypothesize that the reduction of current ART access will result in erasing the success of TasP in controlling the HIV epidemic in the US. We also estimate the outcomes associated with PrEP from 2017 to 2025 and discuss the future increase in ART access needed to sustain the declining incidence trend observed since 2011.

## Methods

### Population

We constructed a deterministic analytical model to assess the outcomes of TasP. We simulated a dynamic cohort of PLHIV, representative for the US, stratified into 4 transmission groups based on the type of contacts each person has: men who have sex with men (MSM), women, heterosexual men, and people who inject drugs (PWID). Within each transmission group, we divided the population based on the HIV care cascade into 4 subgroups: undiagnosed, diagnosed not receiving ART, receiving ART but not virally suppressed with detectable viremia, and virally suppressed (eFigure 4 in Supplement 1). We followed the Consolidated Health Economic Evaluation Reporting Standards (CHEERS) reporting guideline for this analysis. Because this analysis did not involve any human participant data and was not a clinical investigation, the Fred Hutchinson Cancer Center institutional review board did not require approval.

### Transmission Model

For simplicity, we assume that HIV transmissions occur between people in the same transmission group or between heterosexual groups, not allowing for crossover transmissions. In the model, PLHIV can transmit HIV through sexual contacts or through needle sharing (for PWID). We considered 2 types of sexual contacts: stable contacts (recurrent contacts between 2 people) and one-off contacts (occasional or one-time contacts) with all contacts between respective groups being well-mixed. PLHIV with suppressed viral load were assumed to be noninfections (U = U), whereas PLHIV receiving ART with detectable viremia were assumed to experience a 50% reduction in HIV transmission to their partners based on studies linking ART adherence, viral load, and effectiveness from South Africa and US.^10,11^ PLHIV who are diagnosed but not receiving treatment are assumed to be equally as infectious as undiagnosed PLHIV. PrEP use by sexual and needle sharing partners reduces HIV transmission based on preassigned PrEP adherence: high, medium, and low adherence with associated effectiveness per type of transmission contact (sexual or needle sharing).

We used observed annual HIV incidence, prevalence, and care cascade data to calibrate the mean number of new acquisitions generated by each PLHIV every year, accounting for the number of contacts (by transmission group), the type of contact (stable vs occasional) and the per-contact infectivity (heterosexual, homosexual, or through needle sharing) (eFigure 4 in Supplement 1). Model and scenarios were run in Python version 3.10 (Python Software Foundation).

### Alternative Scenarios

We created counterfactual backcasting and forecasting scenarios, discussed individually subsequently, by changing the HIV care cascade and PrEP conditions. We then updated counterfactual HIV prevalence in each transmission group annually as follows:

*Prevalence*(*year_n+_*_1_) = *Prevalence*(*year_n_*) − *Deaths*(*year_n_*) + *Incidence*(*year_n+_*_1_).

Complete model description is provided in the eMethods in Supplement 1, including model equations, parameter values, and corresponding sources and details on model calibration.

### Backcasting

To estimate the outcomes associated with the improvement of the HIV care cascade on the HIV epidemic in US from 2011 to 2025, we first initialized the model to the HIV prevalence and care cascade data at the start of 2011 across the 4 transmission groups considered. Next, we simulated a counterfactual scenario with no improvement in the care cascade, assuming that the proportions of PLHIV in each stage remained fixed at their 2011 levels throughout this period. Finally, we compared this counterfactual scenario with available historical surveillance data for HIV incidence in the same period^12^ and projected the effectiveness of the improving HIV care cascade and in combination with PrEP use after its introduction in 2017.

### Forecasting

To estimate the expected impact of cutting ART subsidies,^10^ we reinitialized the model to the HIV prevalence and care cascade in 2025. We then simulated scenarios in which 10%, 20%, or 30% of PLHIV currently receiving ART lose access immediately, corresponding to the estimated proportion supported by ADAP,^9^ and projected the number of new HIV acquisitions over the next 5 years. ART reduction was applied evenly across transmission groups and among those with detectable viremia and those virally suppressed. Projected cumulative number of HIV acquisitions over the next 5 years (2026-2030) and HIV incidence in 2030 were compared with a conservative status quo scenario assuming no change in the care cascade, maintaining the current proportions of PLHIV who are undiagnosed, diagnosed not receiving treatment, receiving ART with detectable viremia, and virally suppressed, and no further increase in PrEP use. In addition, we explored an optimistic scenario with a modest increase in ART access, assuming that 10% of those diagnosed and not currently receiving ART would gradually start treatment over 5 years. We assumed that among those starting ART, similar proportions of people would achieve viral suppression compared with those already receiving treatment. This scenario resulted in an overall 4% increase in ART use.

### Statistical Analysis

We used the Centers for Disease Control (CDC) database AtlasPlus^12^ as our main data source. The number of deaths among PLHIV and estimated HIV incidence and prevalence stratified by age and transmission group were obtained from AtlasPlus for each year between 2011 and 2025 for which the data were available (different variables are available for different time periods). The number of deaths among PLHIV was obtained for the period of 2010 to 2019 (deaths were not stratified by transmission groups for other years) and was extended using the Lee-Carter model^13^ for the 2020 to 2030 period (eFigure 1 in Supplement 1). HIV prevalence data by transmission and care cascade group was available for the period of 2017 to 2022 and was linearly extended for the periods from 2011 to 2017 and from 2022 to 2025 (eFigure 2 in Supplement 1). Similarly, HIV incidence stratified by transmission group was available for the 2010 to 2022 period, and we linearly estimated incidence from 2022 to 2025 (eFigure 2 in Supplement 1). We assumed that PrEP use increased linearly from 0% in 2017 to the estimated PrEP access by transmission group in 2022 (eTable 1 in Supplement 1) and that these access levels remained constant from 2022 through 2030. eTable 1 in Supplement 1 also describes additional parameters used in the model and corresponding data sources.

## Results

**Figure 1.**
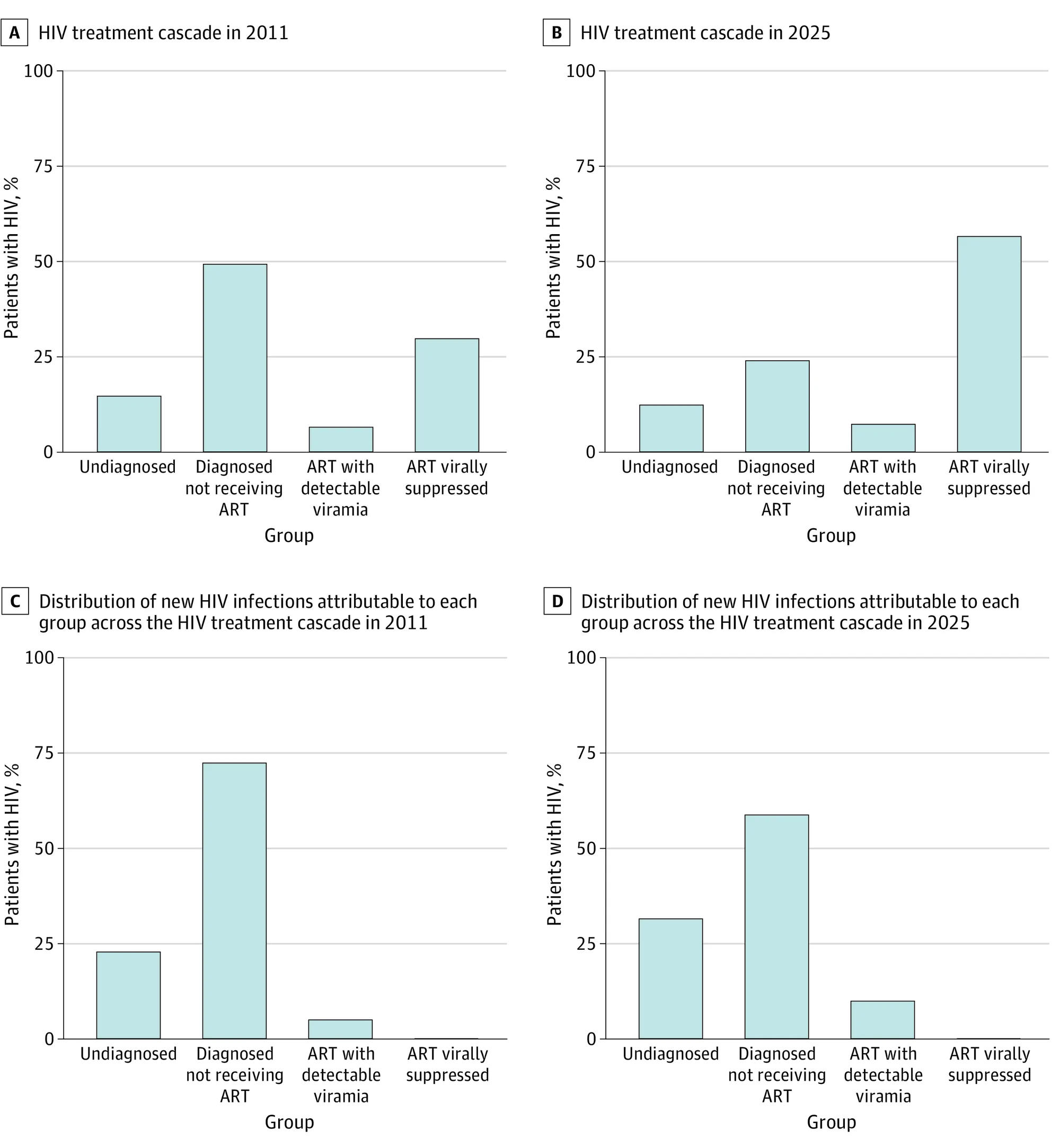
Bar Charts Showing HIV Prevalence and Incidence in 2011 and 2025 Stratified by Treatment Cascade ART indicates antiretroviral therapy.

CDC historical surveillance data^12^ show substantial improvements in the care cascade after TasP was implemented in the US, resulting in increased proportions of PLHIV who were virally suppressed from 29.7% in 2011 to 56.5% in 2025 (Figure 1A and B). This development was associated with a reversal of the previously increasing trend in HIV incidence despite continued growth in the number of PLHIV, reaching 1 238 000 in 2022.^12^

Our results suggest that despite the improvement in care cascade, 58.7% of new HIV acquisitions in 2025 resulted from contact with PLHIV who were diagnosed but not currently receiving treatment, a group representing only 23.9% of PLHIV. In addition, one-third of new acquisitions resulted from contact with undiagnosed PLHIV (Figure 1C and D).

**Figure 2.**
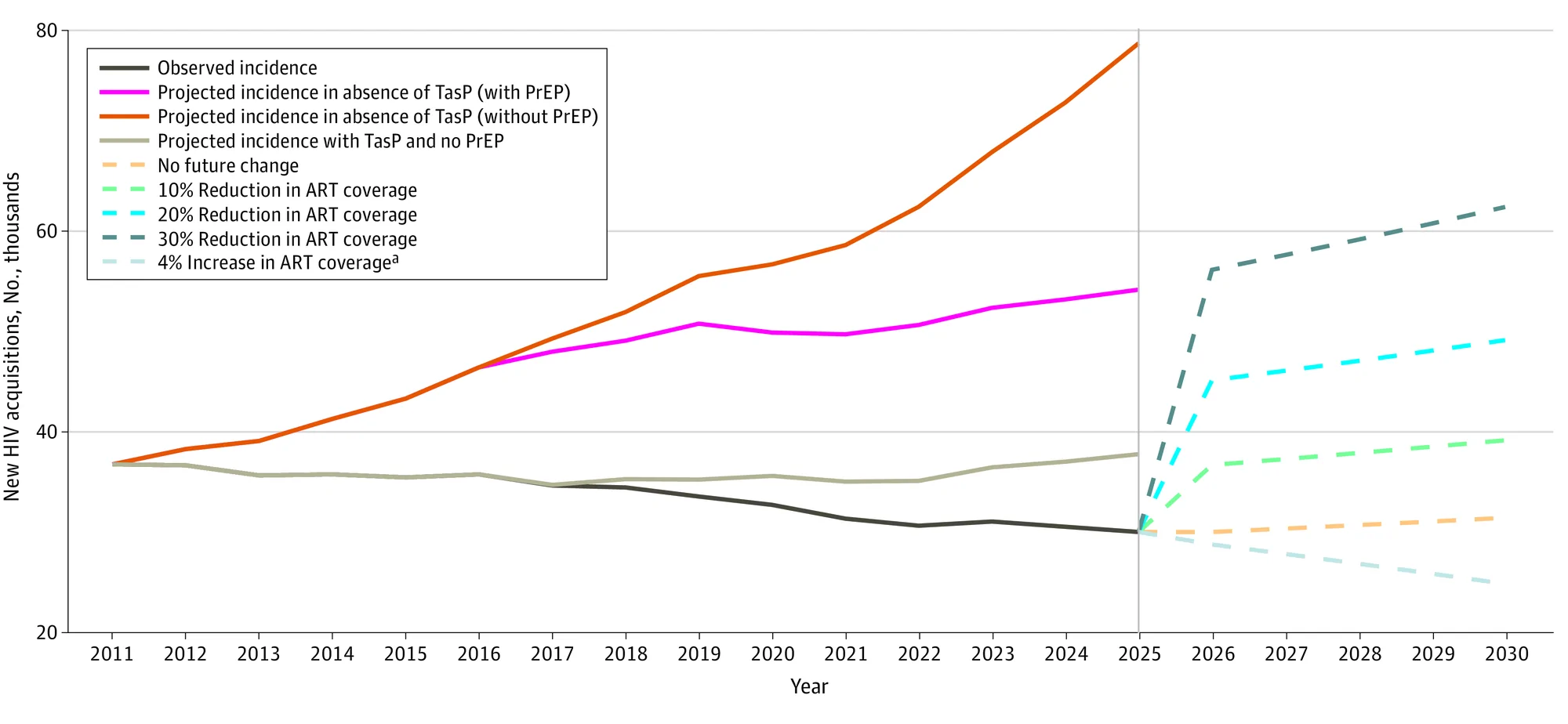
Line Graph Showing Observed vs Estimated HIV Incidence From 2011 to 2025 and Projected HIV Incidence From 2026 to 2030 ART indicates antiretroviral therapy; PrEP, preexposure prophylaxis; TasP, treatment as prevention. ^a^The 4% increase in ART access represents a gradual ART initiation by 10% of those who are diagnosed but not currently in treatment over 5 years.

Our model estimated that TasP alone accounted for a 44.5% reduction in HIV incidence in 2025 (approximately 24 000 new acquisitions prevented) and has prevented approximately 197 400 new HIV acquisitions in the US since 2011, representing a 28.1% reduction over the past 15 years. In the absence of TasP—that is, if the care cascade had remained at its 2011 levels—annual HIV infections in the US would have increased steadily from approximately 36 800 in 2011 to about 49 000 after 2018 (Figure 2, Table 1). The introduction of PrEP in 2017 prevented an estimated 33 200 acquisitions (6.1% of new acquisitions averted) and accounted for a 20.5% reduction in HIV incidence in 2025 (Figure 2, Table 1). These 2 interventions (TasP and PreP) have acted in a synergistic way over the past 9 years, averting approximately 293 000 HIV acquisitions(36.7%) (Figure 2, Table 1).

**Table 1.** Counterfactual and Observed HIV Incidence From 2011 to 2025^a^

Year	Observed incidence, No.	Counterfactual incidence (no TasP, with PreP), No.	Acquisitions averted by TasP, %	Counterfactual incidence (with TasP, no PrEP), No.	Acquisitions averted by PrEP, %	Counterfactual incidence (no TasP, no PreP), No.	Acquisitions averted by TasP and PrEP, %
2011	36 800	36 800	0.0	36 800	0	36 800	0.0
2012	36 700	38 317	4.2	36 700	0	38 317	4.2
2013	35 700	39 122	8.7	35 700	0	39 122	8.7
2014	35 800	41 315	13.3	35 800	0	41 315	13.3
2015	35 500	43 332	18.1	35 500	0	43 332	18.1
2016	35 800	46 438	22.9	35 800	0	46 438	22.9
2017	34 700	47 996	27.7	34 759	0.2	49 303	29.6
2018	34 500	49 092	29.7	35 327	2.3	51 938	33.6
2019	33 600	50 780	33.8	35 284	4.8	55 512	39.5
2020	32 770	49 897	34.3	35 650	8.1	56 677	42.2
2021	31 400	49 726	36.9	35 082	10.5	58 591	46.4
2022	30 700	50 654	39.4	35 155	12.7	62 400	50.8
2023	31 112	52 357	40.6	36 502	14.8	67 842	54.1
2024	30 594	53 192	42.5	37 065	17.5	72 763	58.0
2025	30 075	54 161	44.5	37 811	20.5	78 652	61.8
Cumulative infections	505 751	703 179	28.1	538 935	6.2	799 000	36.7

Abbreviations: PreP, preexposure prophylaxis; TasP, treatment as prevention.

^a^ The counterfactual incidence was calculated by assuming no change in the care cascade from 2011 levels.

Immediate reduction of ART access by 10%, 20%, or 30% in a conservative scenario with no changes in the ART care cascade over the next 5 years (maintaining current proportions of PLHIV diagnosed, linked to care, and virally suppressed) would result in approximately an additional 24 800, 67 000, or 122 300 new HIV acquisitions from 2026 to 2030, respectively, completely erasing the progress made by TasP. Maintaining the current level of ART access would not be enough to prevent annual HIV acquisitions from increasing and would result in 31 500 HIV acquisitions in 2030. Reducing the proportion of diagnosed PLHIV not receiving treatment by 10% over the next 5 years (resulting in 4% increase in the ART access) is needed to continue the current trend of steady decline of the number of new HIV acquisitions to approximately 29 700 by 2030 (Table 2).

**Table 2.** Number of HIV New Acquisitions in 2030 and Cumulative Number of HIV Acquisitions From 2026 to 2030 for a Status Quo Scenario

Scenario type	Access, %	No. of HIV new acquisitions in 2030	No. of cumulative HIV acquisitions 2026-2030	No. of additional cumulative acquisitions 2026-2030
Baseline	0	31 486	153 856	0
Reduced ART access	−10	36 851	178 707	24 852
Reduced ART access	−20	45 992	220 848	66 992
Reduced ART access	−30	58 115	276 219	122 364
Increased ART access	10	29 674	148 576	−5280

Abbreviation: ART, antiretroviral therapy.

## Discussion

The introduction of TasP in 2011 transformed the HIV epidemic by providing PLHIV with an extremely effective and accessible strategy for preventing onward HIV transmission. In this study focused on the US, we estimated that TasP has averted approximately 200 000 HIV infections in the last 14 years and accounted for a 44.5% reduction in new acquisitions in 2025 alone. In several resource-limited countries with very high HIV prevalence, aggressive TasP programs have reduced HIV incidence to near 0 in the general population, confirming the power of this approach.^11,14,15^

Our findings suggest that reductions in ART access in the US would undermine not only the health of individuals who rely on these life-saving treatments, but also more than a decade of progress toward HIV elimination. Such policy changes are likely to lead to thousands of additional HIV acquisitions by reversing gains achieved through improvements across the HIV care cascade. These results are consistent with previous studies that have reported similar increases in HIV incidence under comparable policy scenarios.^16^ Moreover, in the context of a growing population of people living with HIV, continued strengthening of the HIV care cascade will be essential to sustain—and accelerate—the decline in HIV incidence observed over the past 15 years.

### Limitations

This work has several limitations. First, we do not explicitly model different stages of HIV infection. Because the acute phase is the most infectious, this simplifying assumption may lead to an underestimation of new HIV acquisitions resulting from transmission by undiagnosed individuals. However, the prevalence of acute HIV infection among people living with HIV in the US is small (less than 1%); therefore, our main conclusions are unlikely to be affected. We do not consider secondary transmissions arising from new acquisitions. In this respect, our estimates should be considered conservative. Our model does not account for contacts between transmission groups (other than heterosexual contacts). This simplifying assumption was made for 2 reasons: (1) infectivity of insertive contacts among MSM is 20-fold more infectious than all the other contacts, hence HIV transmission is more likely to occur among this type of contact than between MSM and other transmission groups; (2) we did not find reliable data to accurately model these contacts. Limited data on PrEP use introduces uncertainty into our estimates of PrEP’s outcomes. Furthermore, we assumed no changes in PrEP use when access to ART is limited. However, restrictions to ART access could influence both the uptake of and adherence to PrEP as well as risk perception and engagement with prevention services, potentially either offsetting or amplifying the overall impact of reduced treatment availability on HIV transmission. Finally, the loss of funding is state-based, and it will likely affect different populations differently. In this sense, our results should be construed as an upper bound in the number of people losing ART access, and hence in the number of attributable new infections. Nonetheless, we do not believe that these assumptions change our results qualitatively, and furthermore, our results are in good agreement with previously published studies^17,18^ that have reported similar findings.

## Conclusions

Using a decision analytical model to assess the outcomes of reductions in ART access, we estimated that TasP remains one of the most impactful HIV interventions implemented in the US. Cutting ADAP subsidies could lead to thousands of additional infections, substantially undermining the progress made in reducing the HIV burden in the US. Continued strengthening of the HIV treatment cascade, alongside effective prevention strategies such as PrEP, will be essential to achieving HIV elimination in the future.

## Notes

Supplement 1. eMethods. eFigure 1. HIV Prevalance From 2011 to 2025 Stratified by Transmission Group and Care Cascade Stage eFigure 2. Observed vs Simulated HIV Prevalence and Incidence From 2011 to 2025 eFigure 3. Number of Deaths Per Year Between 2011 and 2030 in Each Transmission Group eFigure 4. Model Diagram eTable. Parameter Table eReferences.

Supplement 2. Data Sharing Statement
