# Error in Statistical Analysis

**DOI:** 10.1001/jamanetworkopen.2026.43353

**Published:** 2026-10-07

In the Original Investigation titled “Treatment as Prevention and HIV Transmission in the US,”^1^ published August 21, 2026, there was an error in the Statistical Analysis section. The text should have stated that the data source was the US Centers for Disease Control AtlasPlus database, not the US Centers for Disease Control AidsVu database.^1^ This article has been corrected.
