# Supplementary material for: Treatment as Prevention and HIV Transmission in the US
Source: JAMA Netw Open. 2026 Aug 21;9(8):e2630330. doi: 10.1001/jamanetworkopen.2026.30330 (PMC13498995; doi:10.1001/jamanetworkopen.2026.30330)
Supplement: Supplementary file 1 [file jamanetwopen-e2630330-s001.pdf]

Supplemental Online content.
Matrajt L, Stansfield S, Moore M, et al. Treatment as Prevention for HIV Transmission in the US. JAMA Netw Open. 2026;9(8):e2630330. doi:10.1001/jamanetworkopen.2026.30330
eMethods.
eFigure 1. HIV Prevalence from 2011 to 2025 Stratified by Transmission Group and Care Cascade Stage
eFigure 2.Observed vs Simulated HIV Prevanlence and Incidence From 2011 to 2025
eFigure 3. Number of Deaths Per Year Between 2011 and 2030 in Each Transmission Group
eFigure 4. Model Diagram
eTable. Parameter Table
eReferences
This supplemental material has been provided by the authors to give readers additional information about their work.

1 Methods

We constructed a risk-equation based deterministic mathematical model to assess the impact of treatment as prevention. The model simulates a cohort of people living with HIV (PLHIV) which resembles the population of PLHIV in the United States in 2011. Based on CDC data [1], we stratify PLHIV in four transmission groups based on the type of contacts (MSM, heterosexual men, heterosexual women, and PWID) and care status (Undiagnosed, Diagnosed not on ART, and on ART). People taking ART are further split into two groups: those who are fully virally suppressed, with no detectable viremia and those who have detectable viremia, and hence have partial effectiveness in reducing HIV transmission to their partners.

Transmission: For simplicity, we assume that HIV transmissions occur between people in the same transmission group or between heterosexual groups, that is, we assume no cross-over transmissions. We considered two types of partnerships where HIV transmission can occur: stable contacts (recurrent contacts between two people) and "one-offs" contacts (these are occasional single-time encounters). We assume that contacts between respective groups are homogeneous. We assume that PLHIV can transmit HIV through sexual contacts or through needle sharing (for PWID). Partnerships are calculated on a yearly basis. People who are undiagnosed or diagnosed but not on treatment are assumed to be equally infectious (Figure 4). Based on studies of the connection between ART adherence, viral load and effectiveness from South Africa and U.S. [2, 3], we assume that people with detectable viremia have 50% effectiveness in reducing HIV transmission to their partners.

Model equations: We consider four transmission groups 1-MSM, 2-women, 3-heterosexual men, and 4-PWID) and 4 clinical stage groups (1-undiagnosed, 2-diagnosed not on ART, 3-diagnosed on ART, on ART with detectable viremia, and 4-on ART, virally suppressed): Let r_i be the average number of infections produced by an infected individual from transmission group i with (1-MSM, 2-women, 3-heterosexual men, and 4-PWID), over one year:

$$\begin{aligned} r_i = \quad & (1-\alpha_j)\sum_{j=1}^{4}\sum_{l=1}^{4} d_l\Big\{p_{ij}(1-(1-rel_{il}\beta_{ij})^{a_{ij}}) + q_{ij}rel_{jl}\beta_{ij}\Big\} + \\ & \alpha_j\sum_{j=1}^{4}\sum_{l=1}^{4} d_{jl}\Big\{p_{ij}(1-(1-rel_{il}\epsilon_p\beta_{ij})^{a_{ij}}) + q_{ij}rel_{jl}\epsilon_p\beta_{ij}\Big\} \end{aligned} \tag{1}$$

with

- α_j is the proportion of contacts for group j who are on PrEP,
- d_l is the proportion of the population in transmission group j that is in clinical stage group l,
- p_{ij} are the average number of partnerships between people in transmission group j and uninfected people in transmission group i,
- q_{ij} are the average number of one-time contacts between people in transmission group j and uninfected people in transmission group i,
- rel_{il} is the relative reduction in transmission due to ART in stage group l for people in transmission group i,
- $(1-\epsilon_j)$ is the relative reduction in transmission due to PrEP in transmission group j,
- β_{ij} is the probability of transmission for a contact between a person in transmission group j and uninfected person in transmission group i, and
- a_{ij} average number of contacts between people in transmission group j and uninfected people in transmission group i.

Note that we are assuming no crossover, so equation 1 simplifies as follows for each transmission group:

$$\begin{aligned}
r_2 = &\quad (1-\alpha_3)\sum_{l=1}^4 d_{2l}\Big\{p_{23}(1-(1-rel_{2l}\beta_{23})^{a_{23}}) + q_{23}rel_{2l}\beta_{23}\Big\} + \quad \text{(heterosexual women)}\\
&\quad \alpha_3\sum_{l=1}^4 d_{2l}\Big\{p_{23}(1-(1-rel_{2l}\epsilon_p\beta_{23})^{a_{23}}) + q_{23}rel_{2l}\epsilon_p\beta_{23}\Big\}\\
r_3 = &\quad (1-\alpha_2)\sum_{l=1}^4 d_{3l}\Big\{p_{32}(1-(1-rel_{3l}\beta_{32})^{a_{32}}) + q_{32}rel_{3l}\beta_{32}\Big\} + \quad \text{(heterosexual men)}\\
&\quad \alpha_2\sum_{l=1}^4 d_{3l}\Big\{p_{32}(1-(1-rel_{3l}\epsilon_p\beta_{32})^{a_{32}}) + q_{32}rel_{3l}\epsilon_p\beta_{32}\Big\}\\
r_4 = &\quad (1-\alpha_4)\sum_{l=1}^4 d_{4l}\Big\{p_{44}(1-(1-rel_{4l}\beta_{44})^{a_{44}}) + q_{44}rel_{4l}\beta_{44}\Big\} + \quad \text{(PWID)}\\
&\quad \alpha_4\sum_{l=1}^4 d_{4l}\Big\{p_{44}(1-(1-rel_{4l}\epsilon_p\beta_{44})^{a_{44}}) + q_{44}rel_{4l}\epsilon_p\beta_{44}\Big\}
\end{aligned}$$

We can then compute the expected incidence, denoted by new_j in population j:

$$\begin{aligned}
new_j &= (1-\alpha_i)pop_i\,\bar{d} * \left(p_{ji}(1-(1-\overline{rel} * \beta_{ji})^{a_{ji}}) + q_{ji}\beta_{ji}\overline{rel}\right) + \\
&\alpha pop_i \sum_{k=1}^3 \gamma_k \left(p_{ji}\bar{d} * (1-(1-\epsilon_k\overline{rel}_i * \beta_{ji})^{a_{ji}}) + q_{ji}\epsilon_k\beta_{ji}\bar{d} * \overline{rel}\right)
\end{aligned}$$

where pop_i denotes the population in transmission group i, the vectors $\bar{d} = [d_1, d_2, d_3, d_4]$, $\overline{rel_i} = [rel_{i1}, rel_{i2}, rel_{i3}, rel_{i4}]$ denote the clinical stage distribution in transmission group i and relative reduction in effectiveness according to clinical stage and $*$ denotes the dot product. Given our constraints, we have that if $i = 2$ then $j = 3$, if $i = 3$ then $j = 2$ and $i = j = 4$.

For the MSM group, we compute the average number of infections produced by an infected individual in the MSM group while practicing insertive sex and the average number of new infections produced by an infected individual while practicing receptive sex. Let pop_{ins} and pop_{rec} be the number of MSM living with HIV who were infected due to insertive and receptive sex respectively. Then,

$$\begin{aligned}
pop_{ins} &= \tfrac{\beta_{ins}}{\beta_{ins}+\beta_{rec}} pop_1\\
pop_{rec} &= \tfrac{\beta_{rec}}{\beta_{ins}+\beta_{rec}} pop_1
\end{aligned}$$

where β_{ins} is the probability of male-to-male HIV transmission for insertive sex and β_{ins} is the probability of male-to-male HIV transmission for receptive sex. Analogously, we assume that the number of new infections new_1 among MSM individuals is the result of a weighted average of the number of new infections arising from insertive sex new_{ins} and the number of new infections arising from receptive sex new_{rec}.

$$\begin{aligned}
new_{ins} &= \frac{\beta_{ins}}{\beta_{ins}+\beta_{rec}} * new_1\\
new_{rec} &= \frac{\beta_{rec}}{\beta_{ins}+\beta_{rec}} * new_1
\end{aligned}$$

With these definitions, we compute new_{ins} and new_{rec} similarly to the equations for the groups 2, 3, and 4:

$$\begin{aligned}
new_{rec} &= pop_{ins}\,\bar{d} * \left(p_{11}(1-(1-\overline{rel} * \beta_{rec})^{a_{11}} + q_{11}\beta_{rec}\overline{rel}\right)\\
new_{ins} &= pop_{rec}\,\bar{d} * \left(p_{11}(1-(1-\overline{rel} * \beta_{ins})^{a} + q_{11}\beta_{ins}\overline{rel}\right)
\end{aligned}$$

and

$$new_1 = new_{rec} + new_{ins}.$$

Model Calibration and implementation: The model calibration and implementation was done in several steps.

1. CDC database AidsVu provides estimated prevalence by transmission group and care stage from 2017 to 2022. We additionally found data for 2011 [4]. Using these data sources, we linearly extrapolated the care cascade distributions for each transmission group (Figure 1).

2. We calibrated the number of transmission acts in each transmission group (sexual acts for MSM, heterosexual men and women, needle sharing acts for PWID) using observed (or linearly extrapolated) prevalence to match observed (or extrapolated) incidence. The calibration was done by using an optimization algorithm to fit to the number of sex and needle sharing events in each transmission group to match the incidence in 2011 or in 2025. Simulated incidence and prevalence together with observed incidence and prevalence are shown in Figure 2.

3. Using these calibrated parameters, we ran the model to compute yearly estimates of HIV incidence across transmission and care cascade groups for the different scenarios considered. HIV prevalence was updated using the following formula:

$$\text{Prevalence}[i, j, k] = \text{Prevalence}[i, j, k-1] - \text{Deaths}[i, j, k-1] + \text{Incidence}[i, j, k] \tag{2}$$

where $[i, j, k]$ represents the transmission group i ($i = 1, \ldots, 4$) for MSM, Women, Heterosexual men, and PWID respectively), care stage j ($j = 1, \ldots, 4$ for Undiagnosed, Diagnosed not on ART, On ART with detectable viremia and On ART virally suppressed) and year k (k ranging from 2011 to 2030). For example, for the counterfactual scenario assuming no change in the care cascade from 2011 to 2025, we used the incidence and prevalence of 2011 and computed the number of new acquisitions for 2012. We then used equation 2 to obtain the new prevalence in 2012. We continue in this fashion by estimating yearly incidence and prevalence, without ever changing the proportion of people in each of the care cascade stages. In contrast, to estimate the annual effects of TasP, we follow the same pattern but we update the proportion of people in each of the care stages according to the data.

To estimate the proportion of new acquisitions attributable to each care stage, we rerun the model with the same prevalence and care percentages, but we assume that only those in the group of interest can transmit, while keeping the transmission probabilities of the other groups at zero.

To estimate the effects of PrEP, we followed a similar approach: we simulated a scenario with TasP but without PrEP going from 2011 to 2025, and compared incidence in this scenario with historical incidence.

eFigure 1: HIV Prevalence From 2011 to 2025 stratified by Transmission Group and Care Cascade Stage

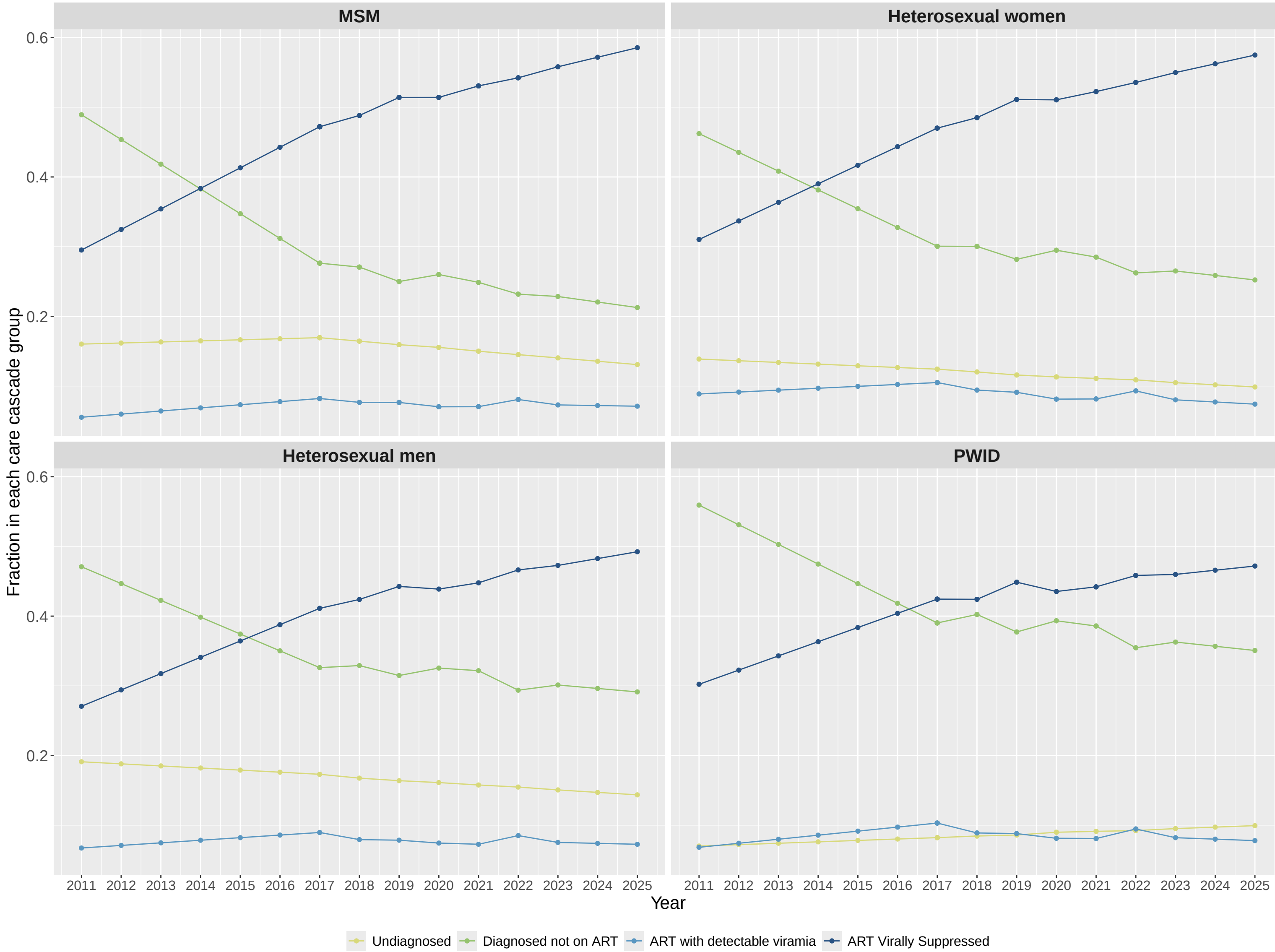

eFigure 2: Observed vs. Simulated HIV Prevalence and Incidence From 2011 to 2025

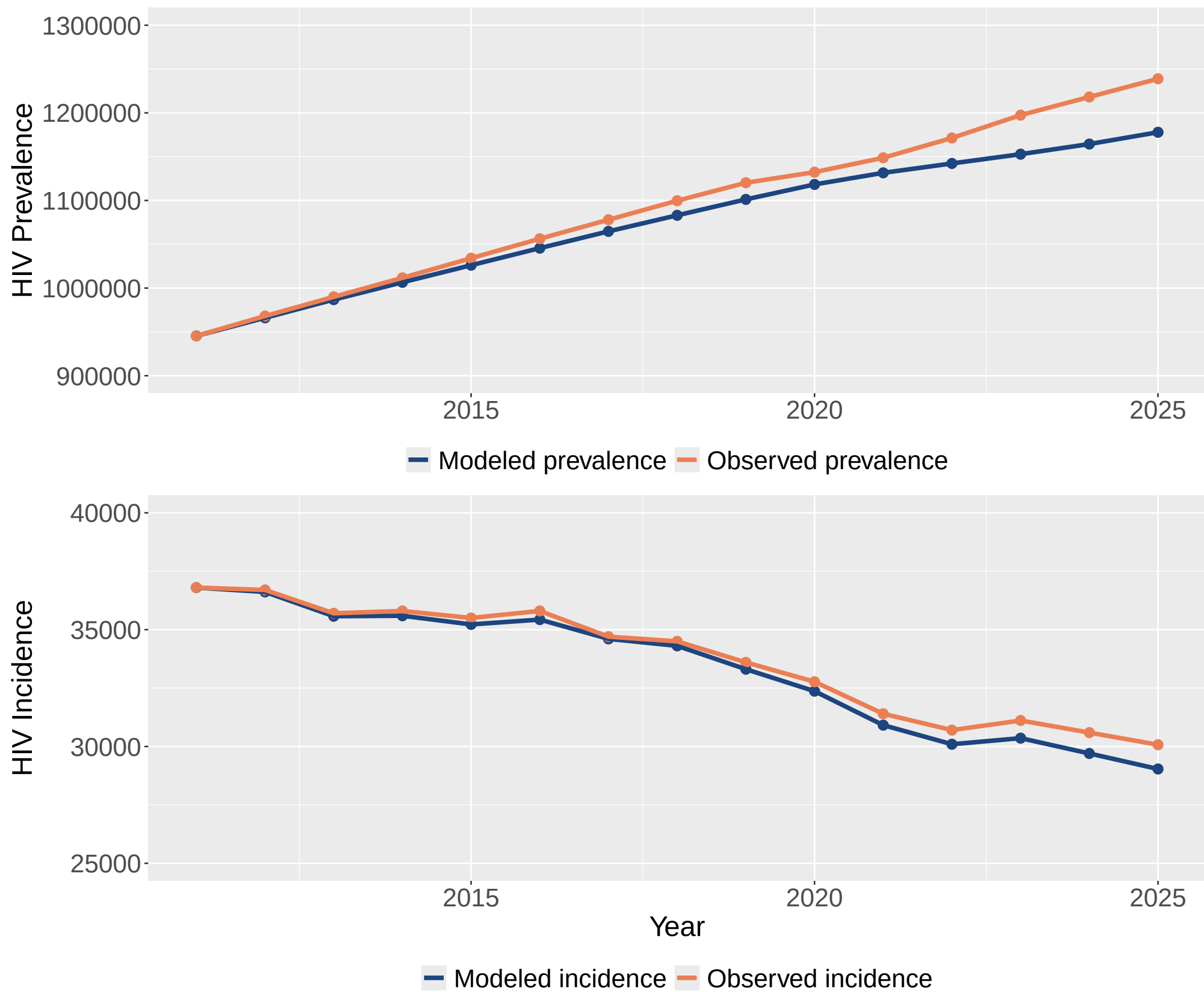

eFigure 3: Number of Deaths Per Year Between 2011 and 2030 in Each Transmission Group.

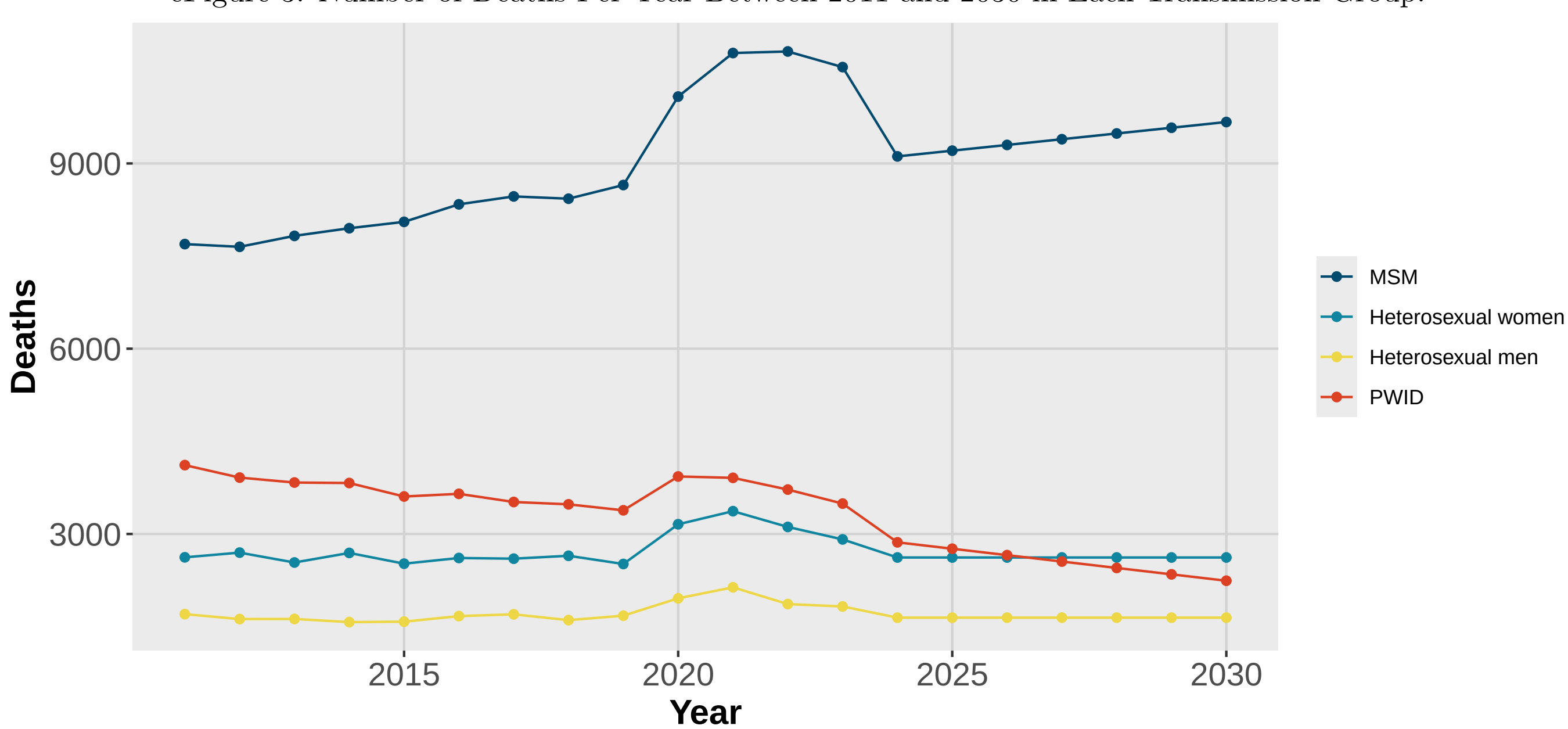

eFigure 4: Model diagram. This figure was created in *http://Biorender.com*

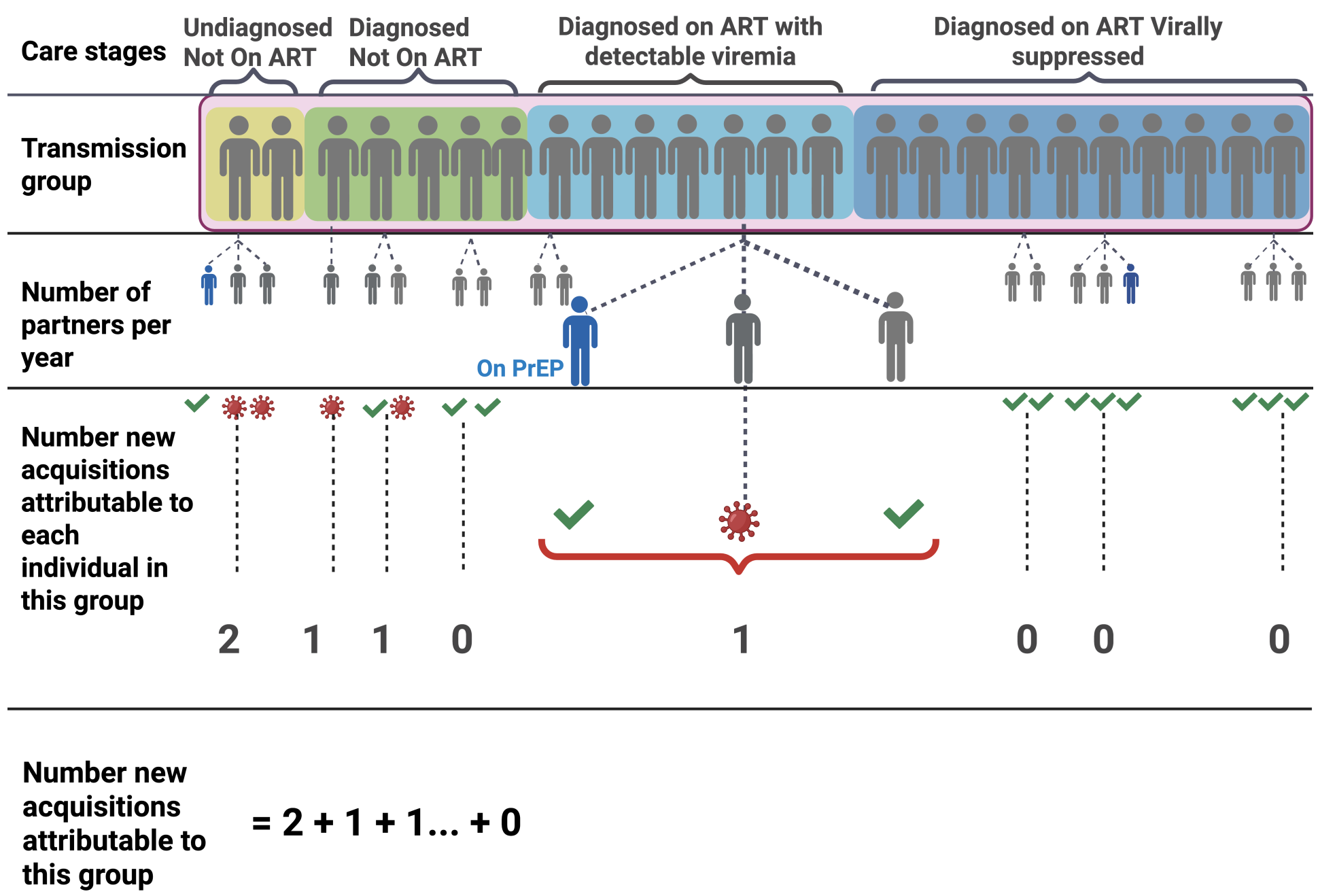

© Matrajt L et al. *JAMA Network Open.*

eTable. Parameter Table

Parameter	Value	Reference
Number of PLHIV in U.S. in 2022	1200000.00	[5]
PLHIV, fraction MSM in 2022	0.65	[5]
PLHIV, fraction heterosexual female in 2022	0.17	[5]
PLHIV, fraction heterosexual male in 2022	0.08	[5]
PLHIV, fraction PWID in 2022	0.10	[5]
HIV incidence, total in 2022	31800.00	[5]
HIV incidence, fraction MSM in 2022	0.71	[5]
HIV incidence, fraction heterosexual female in 2022	0.15	[5]
HIV incidence, fraction heterosexual male in 2022	0.07	[5]
HIV incidence, fraction PWID in 2022	0.07	[5]
Probability of male to female transmission pr act	0.0008	[6]
Probability of injection use	0.01	[7]
Female to male multiplier	0.50	Calculated
Male to male receptive multiplier	24.25	Calculated
Male to male insertive multiplier	1.38	Calculated
Number of annual partnerships, males	0.72	[8]
Number of annual partnerships, females	0.72	[8]
Number of annual partnerships, PWID	2.00	[8]
Number of annual partnerships, MSM receptive	0.95	[8]
Number of annual partnerships, MSM insertive	0.95	[8]
Number of annual one time partnerships, males	0.31	[8]
Number of annual one time partnerships, females	0.31	[8]
Number of annual one time partnerships, MSM receptive	4.16	[8]
Number of annual one time partnerships, MSM insertive	4.16	[8]
Number of annual one time partnerships, PWID	0.00	Assumed
Fraction of MSM insertive	0.22	[8]
Fraction of MSM receptive	0.27	[8]
Fraction of MSM versatile	0.52	[8]
Relative susceptibility for PrEP users among PWID	0.73	[9]
Fraction of PWID receptive syringe HIV+	0.18	[10]
Fraction of PWID receptive syringe HIV-	0.21	[10]
Fraction of PWID distribute syringe HIV+	0.18	[11]
Fraction of PWID distribute syringe HIV-	0.31	[10]
Relative susceptibility for PrEP users, high adherence	0.01	[12]
Relative susceptibility for PrEP users, medium adherence	0.19	[12]
Relative susceptibility for PrEP users, low adherence	0.69	[12]
Fraction of MSM PrEP users who have high adherence	0.78	[13]
Fraction of MSM PrEP users who have medium adherence	0.13	[13]
Fraction of MSM PrEP users who have low adherence	0.09	[13]
Fraction of heterosexual men/women PrEP users who have high adherence	0.66	[14]
Fraction of heterosexual men/women PrEP users who have medium PrEP adherence	0.13	[14]
Fraction of heterosexual men/women PrEP users who have low PrEP adherence	0.21	[14]
Fraction of PWID PrEP users who have high adherence	0.66	[14]
Fraction of PWID PrEP users who have medium adherence	0.13	[14]
Fraction of PWID PrEP users who have low adherence	0.21	[14]
Fraction of MSM using PrEP	0.33	[15]
Fraction of heterosexual women using PrEP	0.01	[16]
Fraction of heterosexual men using PrEP	0.01	[16]
Fraction of PWID using PrEP	0.00	CROI
Fraction of undiagnosed MSM	0.14	[17]
Fraction of MSM diagnosed not in treatment	0.14	[17]
Fraction of MSM diagnosed detectable viremia	0.08	[17]
Fraction of MSM diagnosed virally suppressed	0.58	[17]
Fraction of PWID undiagnosed	0.08	PrEP CHOICE cohort slides
Fraction of PWID diagnosed not in treatment in 2022	0.31	[5]
Fraction of PWID diagnosed with detectable viremia	0.10	PrEP CHOICE cohort slides
Fraction of PWID diagnosed virally suppressed	0.50	PrEP CHOICE cohort slides
Fraction of undiagnosed heterosexual (men or women) in 2022	0.12	[5]
Fraction of diagnosed not in treatment heterosexual (men or women) in 2022	0.19	[5]
Fraction of diagnosed heterosexual men/women with detectable viremia in 2022	0.13	[5]
Fraction of diagnosed heterosexual men/women virally suppressed in 2022	0.56	[5]

© Matrajt L et al. *JAMA Network Open.*

References

[1] Centers for Disease Control and prevention. AtlasPlus - Tables. https://gis.cdc.gov/grasp/nchhstpatlas/tables.html. URL https://gis.cdc.gov/grasp/nchhstpatlas/tables.html. last accessed: 04/03/2026.

[2] Katharina Kranzer, Stephen D. Lawn, Leigh F. Johnson, Linda-Gail Bekker, and Robin Wood. Community viral load and cd4 count distribution among people living with hiv in a south african township: implications for treatment as prevention. *Journal of acquired immune deficiency syndromes (1999)*, 63:498–505, Aug 2013.

[3] Gary Marks, Lytt I. Gardner, Charles E. Rose, Anne Zinski, Richard D. Moore, Susan Holman, Allan E. Rodriguez, Meg Sullivan, and Thomas P. Giordano. Time above 1500 copies: a viral load measure for assessing transmission risk of hiv-positive patients in care. *AIDS (London, England)*, 29:947–54, May 2015.

[4] H. Bradley, H Irene Hall, Richard J Wolitski, Michelle M Van Handel, Amy E Stone, LaFlam Michael, Jacek Skarbinski, Darrel H Higa, Joseph Prejean, Emma Frazier, Roshni Patel, Pian Huang, Qian An, Ruiguang Song, Tian Tang, and Linda A Valleroy. Vital Signs: HIV Diagnosis, Care, and Treatment Among Persons Living with HIV — United States, 2011. *MMWR Morb Mortal Wkly Rep*, 2014.

[5] CDC. HIV Surveillance Supplemental Report: Monitoring Selected National HIV Prevention and Care Objectives by Using HIV Surveillance Data United States and 6 Territories and Freely Associated States, 2022. Technical report, 2024.

[6] Ottawa - Ontario : Public Health Agency of Canada. HIV transmission risk : a summary of the evidence. https://publications.gc.ca/site/eng/434865/publication.html, 2012.

[7] Pragna Patel, Craig B Borkowf, John T Brooks, Arielle Lasry, Amy Lansky, and Jonathan Mermin. Estimating per-act HIV transmission risk: a systematic review. *AIDS*, 28(10):1509–1519, Jun 2014. ISSN 1473-5571 (Electronic); 0269-9370 (Print); 0269-9370 (Linking). doi: 10.1097/QAD.0000000000000298.

[8] Deven T. Hamilton, Karen W. Hoover, Dawn K. Smith, Kevin P. Delaney, Li Yan Wang, Jingjing Li, Tamika Hoyte, Samuel M. Jenness, and Steven M. Goodreau. Achieving the "Ending the HIV Epidemic in the U.S." incidence reduction goals among at-risk populations in the South. *BMC public health*, 23(1):716, April 2023. ISSN 1471-2458. doi: 10.1186/s12889-023-15563-5.

[9] Kachit Choopanya, Michael Martin, Pravan Suntharasamai, Udomsak Sangkum, Philip A Mock, Manoj Leethochawalit, Sithisat Chiamwongpaet, Praphan Kitisin, Pitinan Natrujirote, Somyot Kittimunkong, Rutt Chuachoowong, Roman J Gvetadze, Janet M McNicholl, Lynn A Paxton, Marcel E Curlin, Craig W Hendrix, and Suphak Vanichseni. Antiretroviral prophylaxis for HIV infection in injecting drug users in Bangkok, Thailand (the Bangkok Tenofovir Study): a randomised, double-blind, placebo-controlled phase 3 trial. *Lancet*, 381(9883):2083–2090, Jun 2013. ISSN 1474-547X (Electronic); 0140-6736 (Linking). doi: 10.1016/S0140-6736(13)61127-7.

[10] CDC. HIV Surveillance Special Report: HIV Infection Risk, Prevention, and Testing Behaviors Among Persons Who Inject Drugs National HIV Behavioral Surveillance, 20 U.S. Cities, 2022 Published Date : 02/01/2024. https://stacks.cdc.gov/view/cdc/150464, 02 2024.

[11] CDC. Special Report 35. https://www.cdc.gov/hiv/library/reports/hiv-surveillance.html., February 2024.

[12] Robert M Grant, Peter L Anderson, Vanessa McMahan, Albert Liu, K Rivet Amico, Megha Mehrotra, Sybil Hosek, Carlos Mosquera, Martin Casapia, Orlando Montoya, Susan Buchbinder, Valdilea G Veloso, Kenneth Mayer, Suwat Chariyalertsak, Linda-Gail Bekker, Esper G Kallas, Mauro Schechter, Juan Guanira, Lane Bushman, David N Burns, James F Rooney, and David V Glidden. Uptake of pre-exposure prophylaxis, sexual practices, and HIV incidence in men and transgender women who have sex with men: a cohort study. *Lancet Infect Dis*, 14(9):820–829, Sep 2014. ISSN 1474-4457 (Electronic); 1473-3099 (Print); 1473-3099 (Linking). doi: 10.1016/S1473-3099(14)70847-3.

[13] Albert Y Liu, Stephanie E Cohen, Eric Vittinghoff, Peter L Anderson, Susanne Doblecki-Lewis, Oliver Bacon, Wairimu Chege, Brian S Postle, Tim Matheson, K Rivet Amico, Teri Liegler, M Keith Rawlings, Nikole Trainor, Robert Wilder Blue, Yannine Estrada, Megan E Coleman, Gabriel Cardenas, Daniel J Feaster, Robert Grant, Susan S Philip, Richard Elion, Susan Buchbinder, and Michael A Kolber. Preexposure prophylaxis for hiv infection integrated with municipal- and community-based sexual health services. *JAMA Intern Med*, 176(1):75–84, Jan 2016. ISSN 2168-6114 (Electronic); 2168-6106 (Print); 2168-6106 (Linking). doi: 10.1001/jamainternmed.2015.4683.

[14] Jill Blumenthal, Sonia Jain, Feng He, K Rivet Amico, Ryan Kofron, Eric Ellorin, Jamila K Stockman, Christina Psaros, Gifty M Ntim, Karen Chow, Peter L Anderson, Richard Haubrich, Katya Corado, David J Moore, Sheldon Morris, and Raphael J Landovitz. Results from a Pre-exposure Prophylaxis Demonstration Project for At-risk Cisgender Women in the United States. *Clin Infect Dis*, 73(7):1149–1156, Oct 2021. ISSN 1537-6591 (Electronic); 1058-4838 (Print); 1058-4838 (Linking). doi: 10.1093/cid/ciab328.

[15] CDC. National HIV Behavioral Surveillance (NHBS), 2023. https://www.cdc.gov/hiv-data/nhbs/index.html, 2023.

[16] CDC. National HIV Behavioral Surveillance (NHBS), 2019. https://www.cdc.gov/hiv-data/nhbs/index.html, 2019.

[17] Marc A Pitasi, Linda Beer, Susan Cha, Shacara Johnson Lyons, Angela L Hernandez, Joseph Prejean, Linda A Valleroy, Stacy M Crim, Lindsay Trujillo, Dominique Hardman, Elizabeth M Painter, Jacqueline Petty, Jonathan H Mermin, Demetre C Daskalakis, and H Irene Hall. Vital Signs: HIV Infection, Diagnosis, Treatment, and Prevention Among Gay, Bisexual, and Other Men Who Have Sex with Men - United States, 2010-2019. *MMWR Morb Mortal Wkly Rep*, 70 (48):1669–1675, Dec 2021. ISSN 1545-861X (Electronic); 0149-2195 (Print); 0149-2195 (Linking). doi: 10.15585/mmwr.mm7048e1.
