# Supplementary material for: Treatment as Prevention and HIV Transmission in the US
Source: JAMA Netw Open. 2026 Aug 21;9(8):e2630330. doi: 10.1001/jamanetworkopen.2026.30330 (PMC13498995; doi:10.1001/jamanetworkopen.2026.30330)
Supplement: Supplementary file 2 [file jamanetwopen-e2630330-s002.pdf]

Data Sharing Statement

Matrajt. Treatment as Prevention for HIV Transmission in the US. *JAMA Netw Open*. Published August 21, 2026. doi:10.1001/jamanetworkopen.2026.30330

Data
Data available: Yes
Data types: Other (please specify)
Additional Information: code to run the model will be available upon publication
How to access data: Data is publicly available. Code will be avaiable at github.com/laura-matrajt
When available: With publication

Supporting Documents
Document types: Statistical/analytic code
How to access documents: github.com/laura-matrajt
When available: With publication

Additional Information
Who can access the data: with publication
Types of analyses: code will be available
Mechanisms of data availability: Publicly available
